# Correlation of dual-source computed tomography/dual-energy imaging with pathological grading of lung adenocarcinoma and its clinical value

**DOI:** 10.12669/pjms.336.13320

**Published:** 2017

**Authors:** Haifeng Jiang, Xiao Li

**Affiliations:** 1 Haifeng Jiang, Department of Radiology, Gansu Provincial Hospital, Lanzhou 730000, People’s Republic of China; 2 Xiao Li, Department of Radiology, Gansu Provincial Hospital, Lanzhou 730000, People’s Republic of China

**Keywords:** Dual-source computed tomography, Lung Adenocarcinoma, Pathological grade

## Abstract

**Objective:**

To explore the correlation of dual-source computed tomography (DSCT)/dual-energy imaging with pathological grading of lung adenocarcinoma.

**Methods:**

A total of 47 patients with lung adenocarcinoma were selected. Tissues were histopathologically confirmed by routine DSCT scanning and dual-energy enhanced scanning. Arterial-phase and venous-phase iodine distribution images and single-energy images at 40-190 keV were obtained. The region of interest was outlined to obtain CT values. The iodine concentrations of each tumor in two phases were recorded to calculate normalized iodine concentrations (NICs).

**Results:**

The maximum diameter and minimum diameter of tumors in low differentiation (LD) group were significantly higher than those of high differentiation (HD) group (P<0.05). In LD group, 70.8% of margins were lobulated, which significantly exceeded that of HD group (30.4%) (P<0.05). Besides, 26.1% of patients in HD group were complicated with ground-glass opacity, which was significantly higher than that of LD group (4.2%) (P<0.05). In venous phase, there were significant differences between the two groups at low energy levels (40-70 keV) (P<0.05). At high energy levels (80-190 keV), the CT values of LD group were slightly higher than those of HD group. In arterial and venous phases, NICs of HD group were lower than those of LD group (P>0.05).

**Conclusion:**

HD and LD groups could be predictably distinguished by single-energy images at low energy levels (40-70 keV) in the venous phase. Quantitative analysis of NIC in the venous phase is also valuable for predicting the pathological grade of lung adenocarcinoma.

## INTRODUCTION

Lung cancer, which is one of the most common malignant tumors, has attracted wide attention recently due to high incidence and mortality rates.^1^ Of all lung cancers, lung adenocarcinoma is most common, accounting for 44.5%-46.5%. Meanwhile, the incidence rate of lung adenocarcinoma has been rising annually.^2^ The degree of malignancy of lung adenocarcinoma can be determined by many factors among which pathological grade is an independent index. If the pathological grade of lung adenocarcinoma can be known before surgery in a noninvasive way, it is of great significance to determine the prognosis and to select appropriate treatment methods.

Dual-source computed tomography (DSCT)/dual-energy imaging is a novel technology, with the advantages of fast imaging and low radiation dose. It provides a new multi-parameter diagnosis mode for lesion analysis to allow material separation and identification.^3^ At present, this technology has mainly been used for the preoperative pathological grading of abdominal tumors. Ma et al.^4^ reported that in 23 cases of renal clear cell carcinoma, the normalized iodine concentrations (NICs) of moderately and highly differentiated patients were significantly different from those of lowly and undifferentiated ones. Wu et al.^5^ found that the patients with lowly differentiated gastric cancer had a significantly different NIC from those of moderately and highly differentiated ones in the arterial phase, but the values were similar in the balanced phase. However, this technology has rarely been employed for the preoperative pathological grading of lung cancer hitherto. Non-invasive imaging is preferred to pathological examination in clinical practice. In this study, the correlation between DSCT/dual-energy imaging and the pathological grading of lung adenocarcinoma was analyzed, aiming to determine the degree of malignancy, to guide treatment and to provide a reference for preoperative case selection.

## METHODS

### Baseline clinical data

A total of 47 patients with lung adenocarcinoma treated from December 2013 to December 2015 were enrolled. This study has been approved by the ethics committee of our hospital, and written informed consent has been obtained from all patients.

### Inclusion criteria

1. Chest X-ray or MSCT examination disclosed isolated tumors
2. Patients compliant to CT scan, without allergy to contrast agents
3. Without history of hyperthyroidism, diabetes mellitus, liver and kidney dysfunction or other malignant diseases before examination
4. Without any antitumor therapies before CT examination.

The 47 patients included 29 males and 18 females aged between 31 and 79 years old, 57 on average. All patients were diagnosed as lung adenocarcinoma by surgery and pathological examination, from which 47 tumor foci were obtained. According to the degree of differentiation, lung adenocarcinoma can be classified into high differentiation (HD), moderate differentiation, low differentiation (LD) and non-differentiation. There were 11 HD cases, 12 cases of moderate differentiation, 21 LD cases and 3 cases of non-differentiation. The patients were divided into an HD group comprising HD and moderate differentiation cases, and an LD group comprising LD and non-differentiation cases.

### CT Methods

### DSCT apparatus

The DSCT apparatus included Siemens 64-slice DSCT scanner (Siemens Somatom Definition Flash, Germany), Ulrich Medical high-pressure syringe (Germany) and Siemens Syngo.via Workplace dual-energy processing application (Germany).

### DSCT scanning method and parameters

Plain CT scan and dual-energy dual-phase enhanced scan were conducted from the thoracic entrance to the level of adrenal gland.

Plain CT scanning was performed by single-source scanning technology. Scanning parameters: Tube current of 110 mAs, tube voltage of 120 kVp, attenuation-based on-line modulation of tube current (CARE dose) of 4 D, collimator width of 32×0.6 mm, screw pitch of 1.0, slice thickness and spacing of 8 mm, and X-ray tube rotation time of 0.5 s.

Dual-energy CT enhanced scanning was carried out using dual-source Liver VNC application in the dual-energy scanning mode as well as tubes A and B to collect image information in the same slice simultaneously. Scanning conditions: Matrix of 512×512, tube A of 140 kV and 80 mAs, tube B of 80 kV and 160 mAs, and CARE dose of 4 D. Scanning parameters: Screw pitch of 0.5 mm and probe width of 32×0.6 mm. Reconstruction parameters: Overlapping of 1.0 mm, slice thickness of 1.5 mm, and reconstruction kernel of D26f. Three groups of images were obtained by scanning at 140 kV and 80 kV and through linear fusion (equivalent to 120 kV, fusion factor of 0.3). During enhanced scanning, 100 ml of non-ionic iodine contrast agent (300 mg I/ml iohexol) was injected from the cubital vein by a high-pressure syringe at a flow rate of 3.5 ml/s and a total dose of 1 ml/kg body weight. After injection, 30 ml of normal saline was injected at the same flow rate. Bolus tracking was used to initiate scanning (threshold for triggering arterial scan: 100 HU). Images were collected 25 s and 30 s after injection of contrast agents in the arterial phase and venous phase, respectively.

### Image measurement and analysis

After scanning, raw data were reconstructed into a single-energy spectrum with a slice thickness of 1.5 mm and input into the Dual-Energy software for Siemens DSCT. The lung adenocarcinoma foci confirmed by postoperative pathological examination were observed in the arterial phase and venous phase, respectively, and the single-energy CT value and iodine uptake dose in the same slice were measured. The region of interest (ROI) for parameter analysis was placed at the maximum level of foci, and round or oval ROI was selected. ROI should not be placed at lung tissue, calcified area, ribs, liquefied necrosis or blood sinus. For arterial and venous phases, ROI should be placed in the same position within the same slice to reduce errors. The ROI area should be 1/2~2/3 of the solid area of foci.

### Statistical analysis

All data were expressed as mean ± standard deviation. P<0.05 was considered statistically significant.

## RESULTS

### Maximum and minimum tumor diameters

The maximum diameter and minimum diameter of tumors in the LD group ((4.6±0.35) and (3.8±0.29) cm) were significantly higher than those of the HD group ((3.3±0.26) and (2.7±0.27) cm) (P<0.05), indicating that the differentiation degree of lung adenocarcinoma was correlated with lesion size.

### Tumor margins

In the LD group, 70.8% of the margins were lobulated, which significantly exceeded that of the HD group (30.4%) (P<0.05). The two groups had similar percentages of spiculated margins (P>0.05) (Table-I).

**Table-I:** Tumor margins.

*Tumor margin*		*HD group (n=23)*	*LD group (n=24)*	*χ^2^*	*P*
Lobulated	Yes	7 (30.4)	17 (70.8)	7.671	<0.05
	No	16 (69.6)	7 (29.2)		
Spiculated	Yes	22 (95.7)	21 (87.5)	1.002	>0.05
	No	1 (4.3)	3 (12.5)		

### Complication with ground-glass opacity

In the HD group, 26.1% of patients were complicated with ground-glass opacity, which was significantly higher than that of the LD group (4.2%) (P<0.05).

### Vascular convergence

In the HD group, 30.4% of patients showed vascular convergence, which was similar to that of the LD group (25.0%) (P>0.05).

### CT values in arterial and venous phases at different energy levels

In the arterial phase, the two groups had similar CT values at different energy levels (P>0.05) (Table-II). In the venous phase, there was a significant difference between the two groups at low energy levels (40-70 keV) (P<0.05). At high energy levels (80-190 keV), the CT values of the LD group were slightly higher than those of the HD group (Table-III).

**Table-II:** CT values in the arterial phase at 40-190 keV (HU, x ± s).

*keV*	*HD group*	*LD group*	*t*	*P*
40 keV (HU)	187.7±6.94	195.63±4.73	0.94	0.356
50 keV (HU)	133.6±4.79	140.25±3.42	1.117	0.274
60 keV (HU)	100.75±3.89	106.13±2.96	1.1	0.282
70 keV (HU)	80.46±3.62	85.23±2.85	1.033	0.311
80 keV (HU)	67.45±3.59	71.92±2.86	0.971	0.34
90 keV (HU)	58.82±3.66	63.00±2.91	0.893	0.38
100 keV (HU)	53.51±3.8	56.8±2.95	0.692	0.495
110 keV (HU)	48.36±3.79	52.46±3.00	0.847	0.405
120 keV (HU)	45.23±3.84	49.26±3.03	0.823	0.418
130 keV (HU)	43.00±3.87	46.85±3.07	0.778	0.444
140 keV (HU)	41.30±3.92	45.41±3.09	0.743	0.464
150 keV (HU)	39.72±3.95	43.48±3.10	0.749	0.461
160 keV (HU)	38.9±3.87	42.57±3.12	0.727	0.474
170 keV (HU)	38.07±3.98	41.57±3.13	0.689	0.497
180 keV (HU)	37.7±4.00	40.91±3.15	0.708	0.485
190 keV (HU)	36.80±4.00	40.32±3.15	0.692	0.495

**Table-III:** CT values in the venous phase at 40-190 keV (HU, x±s).

*keV*	*HD group*	*LD group*	*t*	*P*
40 keV (HU)	206.18±5.76	237.7±8.43	3.124	0.004
50 keV (HU)	147.7±4.6	168.6±6.09	2.829	0.009
60 keV (HU)	111.24±4.2	116.72±4.89	2.406	0.023
70 keV (HU)	87.9±4.12	100.7±4.22	2.166	0.039
80 keV (HU)	73.59±4.21	84.10±3.89	1.826	0.079
90 keV (HU)	63.95±4.27	73.04±3.74	1.590	0.124
100 keV (HU)	57.37±4.33	65.40±3.64	1.407	0.171
110 keV (HU)	53.34±4.65	60.01±3.62	1.119	0.273
120 keV (HU)	49.24±4.45	56.00±3.60	1.171	0.252
130 keV (HU)	46.65±4.48	53.05±3.57	1.105	0.279
140 keV (HU)	44.72±4.51	50.80±3.56	1.048	0.304
150 keV (HU)	43.06±4.55	48.9±3.56	1.001	0.326
160 keV (HU)	42.03±4.55	47.69±3.54	0.971	0.34
170 keV (HU)	41.06±4.57	46.59±3.54	0.947	0.352
180 keV (HU)	40.31±4.58	45.72±3.55	0.924	0.364
190 keV (HU)	40.22±4.66	44.99±3.53	0.79	0.437

### NICs in arterial and venous phases

In both arterial and venous phases, NICs of the HD group ((0.22±0.11) and (0.42±0.12) mg/ml) were lower than those of the LD group ((0.23±0.07) and (0.51±0.10) mg/ml), without significant differences (P>0.05).

## DISCUSSION

The diagnostic value of CT technology for lung adenocarcinoma has been widely reported, but the relationships between CT characteristics and the degree of differentiation of pathological tissues remain largely unknown. CT examination is of evident clinical significance to the evaluation of the differentiation degree of tumor before treatment.^6^

The degree of tumor differentiation has been associated with tumor size.^7^ Lobulated margin means rugged edge of tumor, which is caused by vascular or bronchial obstruction in the growth process or by non-uniform growth rates of the tumor. Zhang et al. reported that^8^ there was a significant difference in the proportions of deep lobulated margins of peripheral lung adenocarcinoma between moderate differentiation, HD and LD groups. Besides, the LD group mostly had deep lobulated margins, with high malignancy degree. We herein found that the LD group had significantly more lobulated margins than those of the HD group (P<0.05). Probably, the adenocarcinoma with a high degree of malignancy often contains different histological structures, degrees of cell differentiation and growth rates of tumor edge, easily forming a lobulated margin.

Spiculated margin is often manifested as radial or spiculated changes, an important sign for diagnosing peripheral lung cancer.^9^ However, this study showed that there was no significant difference in the percentage of spiculated margins between HD and LD groups (P>0.05), which may be of no significance to the evaluation of biological behaviors.

Ground-glass opacity is manifested as a slight increase in the peripheral lung density, with ground glass-like changes, and pulmonary vascular texture can be seen in the background, with clear boundary. Fan et al.^10^ found that there was a significant difference in the percentages of ground-glass opacity between moderate differentiation, HD and LD groups (P<0.05). The peripheral lung adenocarcinoma complicated with ground-glass opacity had a higher degree of differentiation. In this study, significantly more patients in the HD group were accompanied by ground-glass opacity than those in the LD group (P<0.05). Therefore, the degree of differentiation was higher in peripheral lung adenocarcinoma complicated with ground-glass opacity.

Vascular convergence, which refers to traction or wrapping of blood vessels around tumors, reflects the changes in the adjacent structures of lung cancer. Zang et al. found that^11^ there was a significant difference in the percentages of vascular convergence between LD and HD groups. In this study, the HD group was slightly more prone to vascular convergence than the LD group (P>0.05).

Remy et al. reported that^12^ different tissues had different energy spectra due to various chemical compositions. The CT decay curves of substances were determined by their chemical compositions. Thus, the differences between CT decay curves of substances can be used to distinguish the chemical compositions of different tissues or organs. The energy spectrum decay curve of the LD group herein was generally slightly higher than that of the HD group in the arterial phase. The CT values at different energy levels were similar (P>0.05). In the venous phase, similarly, the energy spectrum decay curve of the LD group was also higher than that of the HD group. The difference between the LD group and the HD group was statistically significant at low energy levels (40-70 keV) (P<0.05). Accordingly, low energy levels in the venous phase were beneficial to the identification of differentiation degree.

It is well-documented that iodine concentration and NIC can help identify lesions of different pathological types or grades.^13^ NIC of the LD group was higher than that of the HD group in this study, but the difference was not statistically significant (P>0.05). In the venous phase, NIC of the LD group was higher than that of the HD group, without a statistically significant difference either (P>0.05). The results may be attributed to the differences of tumor tissues with different degrees of differentiation in vascular permeability, degree of vascularization and growth patterns. Tumors with different degrees of differentiation had various malignant behaviors, of which LD cancer cells proliferated rapidly, forming more microvessels. New blood vessels were immature and LD, and abnormal ones in tumor tissues increased correspondingly, leading to the higher iodine concentration in the LD group. Hence, NIC was higher at a lower degree of differentiation.

In summary, we preliminarily explored the correlations between parameters of DSCT/dual-energy imaging such as energy spectra and NIC and the differentiation degree of lung adenocarcinoma. The findings provide valuable evidence for early diagnosis, design of clinical treatment regimens and evaluation of prognosis, and for determining the pathological differentiation degrees of tumor tissues.

### Authors’ contributions

**XL** designed this study and significantly revised the manuscript.

**HJ** performed this study and drafted this manuscript.
